# Uptake of Shingles, Influenza, COVID-19 and Pneumococcal Vaccination in Patients with Inflammatory Arthritis: A Three-Centre Study

**DOI:** 10.3390/vaccines14050400

**Published:** 2026-04-29

**Authors:** Krishika Balakrishnan, Lozan Hussein Mahmood Zangana, Moyinoluwa Rachel Ajayi, Marcin Kowalczyk, Deepak Nagra, Su Li Goh, Mariam Baghaffar, Madusha Jayesinghe, Rofaida Hassan, Asad Khan, Mary Gayed, Alexandra Godlee, Sophia Khan, Sujata Ganguly, Arvind Sinha, Eleni Stathopoulou, Maryam Adas, Zijing Yang, James Galloway

**Affiliations:** 1 University Hospitals Birmingham NHS Trust, Solihull, Heartlands & Good Hope Hospital, Birmingham B75 7RR, UK; bkrishika@gmail.com (K.B.); moyinoluwarachel.ajayi@uhb.nhs.uk (M.R.A.); marcinpiotr.kowalczyk@uhb.nhs.uk (M.K.); suli.goh@uhb.nhs.uk (S.L.G.); mariam.baghaffar@uhb.nhs.uk (M.B.); madusha.jayasinghe@wales.nhs.uk (M.J.); rofaida.hassan@uhb.nhs.uk (R.H.); asad.khan3@uhb.nhs.uk (A.K.); mary.gayed@uhb.nhs.uk (M.G.); alexandra.godlee@uhb.nhs.uk (A.G.); sophia.khan@uhb.nhs.uk (S.K.); sujata.ganguly@uhb.nhs.uk (S.G.); arvind.sinha@uhb.nhs.uk (A.S.);; 2 Centre for Rheumatic Disease, King’s College London, London WC2R 2LS, UK; maryam.adas@kcl.ac.uk (M.A.);; 3 University Hospitals Coventry & Warwickshire NHS Trust, Coventry CV2 2DX, UK

**Keywords:** vaccine, vaccine hesitancy, shingles, pneumococcal, COVID, influenza, rheumatic disease

## Abstract

**Introduction:** Patients with inflammatory arthritis are at increased risk of infection due to immune dysregulation and immunosuppressive therapy. National and international guidelines recommend vaccination against pneumococcal disease, influenza, COVID-19, and herpes zoster; however, uptake remains inconsistent. This study evaluated op-world uptake of multiple recommended vaccines within a large UK cohort. **Methods:** We conducted a cross-sectional study of adults with rheumatoid arthritis, psoriatic arthritis, or ankylosing spondylitis across three hospital sites serving ~800,000 people. Eligible patients had a healthcare encounter within 12 months prior to 1 January 2026. Vaccination status (pneumococcal, influenza, COVID-19, shingles) was obtained from linked primary care records. Demographic and clinical variables were collected. Uptake was reported as percentages with 95% confidence intervals. Associations with pneumococcal vaccination were assessed using Poisson regression with robust standard errors. **Results:** Among 2158 patients (median age 58 years; 72% female), rheumatoid arthritis was most common (61%). Most were receiving biologic or targeted synthetic DMARDs. Vaccine availability was not limited. Uptake was suboptimal: pneumococcal 30%, influenza 29%, COVID-19 53%, and shingles 12%. Pneumococcal uptake was higher in those aged ≥65 years. Increasing age (aRR 1.92, 95% CI 1.52–2.42) and at-risk comorbidities (aRR 1.42, 95% CI 1.20–1.69) were associated with higher uptake, while biologic or targeted therapy was associated with lower uptake (aRR 0.55, 95% CI 0.48–0.63). **Discussion:** Vaccination uptake remains suboptimal in this high-risk population. Lower uptake in patients on advanced therapies highlights a gap in care. Targeted education and better integration of vaccination pathways within rheumatology services are needed.

## 1. Introduction

Vaccination throughout time has been demonstrated to be the single most effective preventative measure against infection, in both healthy individuals and those affected by autoimmune rheumatic diseases (ARD). Those diagnosed with an inflammatory arthritis, such as rheumatoid arthritis, remain at heightened risk of opportunistic infections due to dysregulation of the immune system, but also secondary to treatment [1]. These infections remain preventable with non-live vaccines without the need to completely withdraw treatment to facilitate vaccination. Vaccine design and efficacy has improved over the years since the first smallpox vaccine [2]. All major national and international guidelines for the management of rheumatic disease recommend that patients with inflammatory arthritis should receive pneumococcal, influenza, COVID-19 and shingles vaccination [3,4,5]; however, despite these recommendations, vaccination rates remain substandard. In addition to this, these vaccines are recommended to those without ARDs, for example, the Centre for Disease Control (CDC) recommends COVID-19 vaccination in those over the age of 65 or if any additional risk factors, vaccination between ages 6 months to 64 years. One major predictor in vaccine uptake amongst these cohorts is vaccination advice from healthcare professionals, which is heterogenous [6,7].

Herpes zoster reactivation clinically manifests as shingles causing a considerable symptom burden with pain. Initially approved in 2013, the live attenuated Zostavax was available to those aged over 70 years [8]. However, the vaccine remains unsuitable for those immunosuppressed. More recently, the Shingrix vaccine programme was launched in 2021 comprising two doses of the non-live subunit vaccine. With the initial results published over a decade ago [9], both efficacy and safety concerns are favourable in those with an inflammatory arthritis, however vaccine uptake remains suboptimal [10,11].

Comparatively, the influenza vaccine, since its inception in the 1930s, has been available in both live and non-live formulations for over two decades [12]. Vaccination uptake has fluctuated over the years, with both immunosuppressed and non-immunosuppressed individuals demonstrating variable uptake, at best of around 50% per vaccination cycle [13,14].

A similar theme follows pneumococcal vaccination, which dropped following the COVID-19 pandemic, thought to be a direct result of vaccine hesitancy [15,16,17]. This has been demonstrated not only in healthy, not at-risk individuals but also in those with significant risk factors for invasive pneumococcal disease [18,19]. COVID-19 vaccination, initially mandated by most governments across the globe in the immediate period following the pandemic, has also waned [20] providing a cause for concern.

Few studies have demonstrated differences in a single population assessing vaccine uptake for all four recommended vaccines in those at risk with inflammatory arthritis [4,21]. This study assessed vaccination uptake of shingles, pneumococcal, influenza and COVID-19 in those with inflammatory arthritis who were immunosuppressed in three hospital sites serving a population of 800,000 patients in the United Kingdom.

## 2. Methods

### 2.1. Study Population and Primary Outcome

We performed a cross sectional study assessing vaccine uptake in those with an inflammatory arthritis (rheumatoid arthritis, psoriatic arthritis, or ankylosing spondylitis) across three separate hospital sites serving a population of approximately 800,000 people. The hospitals included were Solihull, Good Hope and Heartlands, University Hospitals Birmingham NHS Trust, UK. Patients must have had an encounter with a healthcare professional in the preceding 12 months from 1 January 2026, prior to data collection, to be eligible. Patients were aged 18 or above, with receipt of the vaccine after diagnosis. Vaccines included were the polysaccharide pneumococcal vaccine (PPSV23), shingles vaccines (Shingrix or Zostavax) at any time point plus influenza and COVID-19 in the last vaccination season. Childhood vaccines were excluded from the analysis.

The primary outcome was receipt of pneumococcal, shingles, COVID-19 or influenza vaccines. Vaccine data were extracted from linked primary care records containing information on vaccine prescriptions in both primary and secondary care. Analysis were performed in R version 4.4.1.

### 2.2. Covariates

Baseline data were collected for age, gender, and comorbidities (asplenia, chronic respiratory, cardiovascular, renal or liver disease and diabetes mellitus). Rheumatic diagnosis and therapy with either conventional (csDMARD), biologic (bDMARD) or targeted synthetic (tsDMARD) disease-modifying anti-rheumatic drugs were recorded. Data were collected by five clinicians.

The season was classified according to the UK Meteorological Office definition. Winter was defined as 1 December to 28 February of the subsequent year, spring as 1 March to 31 May, summer as 1 June to 31 August, and autumn as 1 September to 30 November.

### 2.3. Statistical Analysis

The percentage and 95% CI of the study population were calculated. The proportion vaccinated was further stratified according to age (<65, ≥65 years), sex, type of rheumatic diagnosis, b/tsDMARD status and presence of additional at-risk condition for vaccination, including asplenia, chronic respiratory, cardiovascular, renal or liver disease and diabetes mellitus.

Poisson regression models with robust standard errors were fitted to estimate mutually adjusted associations between pneumococcal vaccination and age group, sex, inflammatory arthritis subtype, and the presence of additional conditions conferring eligibility for vaccination. Multiple imputation with chained equations (*n* = 40) was used to impute missing variables. We calculated and combined adjusted risk ratios (RRs) and 95% CIs using Rubin’s rule across the imputed datasets. All statistical analyses were performed using R, version 4.4.1. The *p* values were from 2-sided tests, and results were deemed statistically significant at a level of *p* < 0.05.

No formal a priori sample size calculation was performed, as the study population was defined by the total number of eligible patients identified across three hospital sites within the study period. The precision of the vaccination uptake estimates was assessed through the width of the 95% confidence intervals, indicating that the achieved sample of 2158 patients was sufficient for the primary descriptive objectives of this study. For the multivariable Poisson regression analyses, the sample provided a sufficient number of observations to support stable estimation of adjusted associations between vaccination uptake and the six covariates of interest. 

### 2.4. Ethics

Approval was granted under the local review board approval for an audit and quality improvement project to identify gaps in vaccine uptake in those who are vulnerable to opportunistic infections. The secondary use of anonymised data for the study was in line with the health research authority (HRA) decision tool and guidance of the National Research Ethics Service (NRSES) and UK Medical Research Council (MRC) research ethics committee (REC) review tool which confirmed ethics approval was not required for this project.

## 3. Results

### 3.1. Patient Characteristics

Data was available for 2158 patients. The average median age of the patients was 58 (IQR 45–68) and 1544 (72%) were female; 796 (36.9%) were white British, with 184 (8.5%) Asian and 30 patients of Afro-Caribbean ethnicity (1.4%). Ethnicity was unreported in 1107 (51.3%) of the patients. Rheumatoid arthritis was the most common diagnosis in 1317 (61%) followed by psoriatic arthritis in 413 (19%), ankylosing spondylitis in 322 (15%) with the remaining patients having an undifferentiated inflammatory arthritis (Table 1).

Amongst treatment regimes, 1560 (72%) were medicated with both csDMARD and bDMARDs of which tumour necrosis factor (TNFi) inhibitors were the commonest 1456 (67%) followed by Janus Kinase inhibitors (JAKi) in 146 (6.8%) and Interleukin-17 (IL-17) inhibitors in 126 (5.8%). Other biologics included Interleukin-6 (IL-6)inhibitors in 81 (3.8%) of patients and Interleukin-23 (IL-23) inhibitors in 11 (0.5%) of patients.

### 3.2. Vaccine Uptake

Overall uptake of pneumococcal vaccination was 30% (*n* = 650, 95% CI 28–32%), influenza vaccine was 29% (*n* = 625, 95% CI 27–31%), COVID-19 vaccine was 53% (*n* = 1151, 95% CI 51–55%) and Shingles vaccine was 12% (*n* = 261, 95% CI 11–14%). Vaccination details are shown in Table 2.

**Figure 1:**
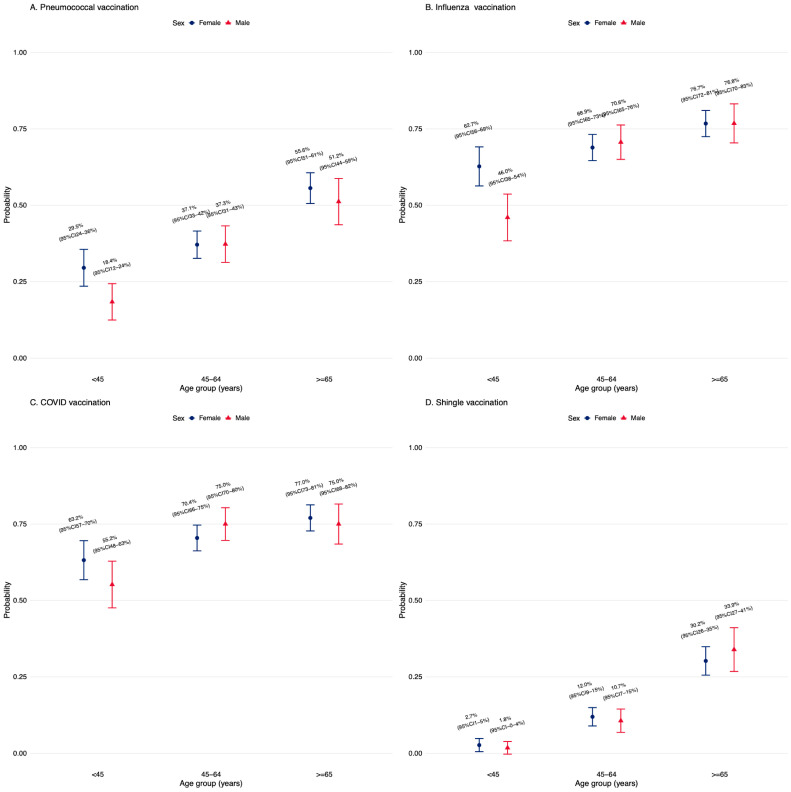
Vaccination across age and gender. Vaccination uptake increased over time across all age groups in both males (blue) and females (red). Uptake was lowest at baseline in younger age groups and highest in older age groups throughout. In all panels, point estimates for males and females were similar, with overlapping error bars at most time points, indicating no consistent gender-based differences. Although males appeared to have marginally higher uptake at earlier time points in some age groups, these differences were not sustained. Error bars represent 95% CIs.

Pneumococcal vaccination uptake was 45.9% (294 of 542; 95% CI, 41.8–50.1%) among patients aged 65 years or older and 32.8% (356 of 1085; 95% CI, 30.1–35.7%) among those younger than 65 years. Among all age and sex strata, women aged 65 years or older had the highest uptake (55.6%; 95% CI, 50.8–60.7%). The probability of receiving the pneumococcal vaccine across age and sex is shown in Figure 1. Among individuals who received pneumococcal vaccination, uptake was highest in autumn (290 [44.6%]), followed by winter (133 [20.5%]), spring (120 [18.5%]), and summer (106 [16.3%]).

Pneumococcal vaccination uptake varied by diagnosis: 33.3% (438/1317; 95% CI, 30.8–35.8%) in rheumatoid arthritis, 21.7% (70/322; 95% CI, 17.5–26.5%) in ankylosing spondylitis, and 28.3% (117/413; 95% CI, 24.2–32.8%) in psoriatic arthritis. In the rheumatoid arthritis subgroup (*n* = 1317), uptake of influenza, COVID-19, and herpes zoster vaccines was 55.0% (725/1317; 95% CI, 52.4–57.7%), 55.3% (728/1317; 95% CI, 52.6–57.9%), and 15.2% (200/1317; 95% CI, 13.3–17.2%), respectively.

Among patient younger than 65 years (*n* = 1086), pneumococcal vaccination uptake was 32.8% (356 of 1086; 95% CI, 30.0–35.6%), influenza vaccination uptake was 64.5% (701 of 1086; 95% CI, 61.7–67.4%), COVID-19 vaccination uptake was 67.8% (736 of 1086; 95% CI, 64.9–70.5%), and shingles vaccination uptake was 8.3% (90 of 1086; 95% CI, 6.8–10.1%). Pneumococcal vaccination uptake among women younger than 65 years (*n* = 670) was 34.6% (232 of 670; 95% CI, 31.1–38.3%). Additional details for this age group are provided in Table S1.

### 3.3. Vaccine Uptake by Risk Factors

Increasing age and the presence of additional at-risk conditions were independently associated with pneumococcal vaccination uptake (Table 3). Vaccination uptake was substantially higher among individuals aged ≥65 years compared with those aged <45 years (adjusted risk ratio [aRR], 1.92; 95% CI, 1.52–2.42), and among those with any at-risk condition compared with those without (aRR, 1.42; 95% CI, 120–1.69). In contrast, exposure to biologic or targeted synthetic DMARDs was independently associated with lower uptake (aRR, 0.55; 95% CI, 0.48–0.63). After adjustment, sex, ethnicity, and inflammatory arthritis subtype were not independently associated with vaccination uptake.

## 4. Discussion

Amongst our cohort of 2158 patients with a diagnosis of an inflammatory arthritis on biologic therapy, in which all patients were eligible for pneumococcal, influenza, COVID-19 and shingles vaccines, the collective receipt of these vaccines was suboptimal. Vaccination rates were higher in the autumn when compared to the summer months, primarily driven by vaccination campaigns through government policy to reduce infection-related hospital admissions during the winter months.

COVID-19 vaccine receipt was highest when compared to other vaccines, however, this was only 53%. Despite a national campaign to promote COVID-19 vaccination uptake since the pandemic, vaccination acceptance has been waning. When compared to other studies, vaccination receipt in our cohort is lower than the described uptake in national registry data. Given that vaccination rates remain suboptimal, this study highlights that patients with ARDs remain at risk of preventable infectious disease.

One striking difference to previous studies within our study was the response of the vaccine by risk factors. In the current study, exposure to biologics was independently associated with lower uptake of vaccination. Previous studies have demonstrated increasing vaccine rates in those on biologics [15]. The study was unable to capture those on monotherapy csDMARD therapy, and this may have confounded this result, and explains our findings.

Limitations exist within this study. Only patients who had contact with a healthcare provider were captured within this study. Furthermore, those on monotherapy csDMARDs were not captured in this study due to the study design. There was no comparator group in ‘at-risk’ individuals with other autoimmune disease for example, inflammatory bowel disease (IBD) or multiple sclerosis (MS). Another limitation was that we were unable to accurately determine the duration of disease in these individuals, a key factor in determining vaccine acceptance. The single most important risk factor would be prior infection or disease to the individual organisms. However, we were not able to capture this data due to heterogenicity of disease coding amongst healthcare staff [22,23]. Furthermore, we were unable to perform a robust statistical analysis on shingles vaccine data due to few patients being vaccinated.

However, the strengths of this study dominate with up-to-date real-world evidence to address the gaps in public health needs in a large urban city prone to communicable disease. Within the rheumatology service, this provides the data to allow for service development, a core principle in healthcare [24]. The study was able to capture a range of biologics, with those at highest risk of shingles with the JAK inhibitors in comparison to monoclonals with low infection risk, such as the IL-23 class.

The UK Health Security Agency (UKHSA) reported the results for the 2024–2025 Shingrix vaccination programme in October 2025. This report highlighted poor vaccination uptake, with single dose uptake reported as 16.2% in the first quarter in those aged 65 or above. When comparing regions, the East of England had the highest uptake, representing 19.3% of all uptake, with London being the lowest demonstrating 9.2% uptake. In a similar study using Clinical Practice Research Datalink (CPRD) over a 3-year period from 2021 when Shingrix was licenced until 2023, Shingrix vaccination in those who were immunosuppressed (86,197 individuals) was 17.3% [8]. Since the introduction of the Shingrix vaccine programme for those at highest risk, no single study has been able to demonstrate a significant vaccination acceptance rate for the Shingrix vaccine, with most studies showing acceptance of between 10 and 20%.

Other studies have previously mirrored similar results, demonstrating suboptimal vaccination uptake in high-risk groups [15,25]. The theme extends beyond ARDs to those who have had transplants and patients undergoing chemotherapy [26,27]. In the studies with cancer patients, over half the cohorts expressed vaccine hesitancy for COVID-19 vaccination [28]. This trend follows with pneumococcal vaccination uptake reported as 56% in studies with solid organ transplant recipients [29]. Despite no substantial safety concerns in these other at risk groups [30,31], vaccination rates remain universally low. Studies from Japan assessing vaccination uptake in those with other comorbidities beyond ARDs have demonstrated similar results with poor uptake yet again. One common concern from patients receiving vaccines has been the need to pause immunosuppression risking a flare of their disease [32]; however, in this Japanese study—including non-immunosuppressed patients—vaccine rates remain poor, suggesting a wider behavioral public health trend [33].

Solutions have been explored, including improving education and access to vaccination [34,35]. Vaccination reminders have been suggested to be amongst one of seven key drivers for vaccine success, an easy but often overlooked intervention.

Various reasons may account for poor uptake, and our findings mirror that of the national data linkage studies. Following the COVID-19 pandemic, vaccine uptake across all age groups and indications has been waning due to mistrust in the vaccine development process following the adverse events with the AstraZeneca vaccine [36,37]. A similar distrust has developed towards the influenza vaccine, with questions about its design and integrity [38]. Furthermore, healthcare workers have demonstrated vaccine hesitancy, questioning whether education programmes are the solution to vaccine hesitancy or not [39,40,41,42,43]. More recently, there have been reports of IgG4 class switching in those receiving multiple doses of COVID-19 vaccination, which in some studies has been associated with increased infection risk. The exact correlation of vaccination frequency and infection risk remains unclear [44,45,46].

Pneumococcal vaccination rates have also shown a decline within the UK over the past 5 years. In a nationwide study within the UK of over two million people, pneumococcal vaccination rates were just over 50% [19] with similar findings in immunosuppressed groups at the highest risk for infection [15,18]. However, despite the vaccine hesitancy following the COVID-19 pandemic, pneumococcal vaccine acceptance was suboptimal in the years prior to the pandemic with 32% of patients receiving the polysaccharide vaccine in a 4-year follow-up study [15]. The JCVI Green book is published by the UK Health Security Agency highlighting vaccination recommendations for those of varying ages living in the United Kingdom. Amongst this cohort, all the patients had a JCVI Green book risk factor for vaccination, and the data were collected between 2011 and 2015. The rates of pneumococcal vaccine receipt were similar to those in our study. Previous studies have highlighted various socioeconomic and psychosocial determinants to reason for vaccine hesitancy, including religious reasons, personal beliefs and safety concerns [47,48,49]. It is likely that the COVID-19 pandemic has further fuelled such beliefs, with an NHS England report published in 2021 confirming these perceived social implications.

Education programmes to date have shown good utility in the uptake of various vaccines [50]. The mode of education has been debated upon, with some studies suggesting public health education should start in school [51,52,53]. The former Education Minister in the UK, Nadhim Zahwai explored vaccine hesitancy and mistrust in late 2021 following the COVID-19 pandemic however vaccine education is not yet part of a formal curriculum in the UK.

## 5. Conclusions 

Despite the positive public health benefits of vaccination, acceptance remains suboptimal, and without health education and promotion, vaccine uptake is likely to continue to wane. Following the results of our study, a vaccination promotion campaign is underway with resources for patients available after each clinic visit with letters to patients to highlight vaccination schedules, vaccination reminders and patient liaison group meetings. Furthermore, a departmental website for patients to access information is being created to provide educational content on vaccination.

Engagement through patient advocacy groups and addressing socio-economic hurdles to vaccination is paramount to improving delivery of the vaccination programmes to reduce preventable opportunistic infections in populations, with increasing morbidity to reduce mortality.

## Figures and Tables

**Table 1:** Baseline characteristics of patients.

Characteristic	Overall *N* = 2158	Pneumococcal Vaccination No *N* = 1508	Pneumococcal Vaccination Yes *N* = 650
**Age**	58 (45, 68)	55 (43, 65)	63 (53, 72)
**Gender-Female,** ***n*** **(%)**	1544 (72%)	1104 (73%)	440 (68%)
**Ethnicity,** ***n*** **(%)**			
White	796 (37%)	424 (28%)	372 (57%)
Asian	184 (8.5%)	106 (7.0%)	78 (12%)
Black	30 (1.4%)	17 (1.1%)	13 (2.0%)
Mixed	18 (0.8%)	14 (0.9%)	4 (0.6%)
Other	23 (1.1%)	14 (0.9%)	9 (1.4%)
Unknown	1107 (51%)	933 (62%)	174 (27%)
**Inflammatory arthritis,** ***n*** **(%)**			
RA	1317 (61%)	879 (58%)	438 (67%)
AS	322 (15%)	252 (17%)	70 (11%)
PSA	413 (19%)	296 (20%)	117 (18%)
AxSp	53 (2.5%)	43 (2.9%)	10 (1.5%)
Undifferentiated IA	53 (2.5%)	38 (2.5%)	15 (2.3%)
**At-risk condition (any),** ***n*** **(%)**	657 (30%)	304 (20%)	353 (54%)
**Cancer,** ***n*** **(%)**	86 (4.0%)	43 (2.9%)	43 (6.6%)
No	1196 (55%)	671 (44%)	525 (81%)
Unknown	876 (41%)	794 (53%)	82 (13%)
**Diabetes,** ***n*** **(%)**	165 (7.6%)	66 (4.4%)	99 (15%)
No	1113 (52%)	642 (43%)	471 (72%)
Unknown	880 (41%)	800 (53%)	80 (12%)
**IHD,** ***n*** **(%)**	79 (3.7%)	33 (2.2%)	46 (7.1%)
No	1203 (56%)	681 (45%)	522 (80%)
Unknown	876 (41%)	794 (53%)	82 (13%)
**Hypertension,** ***n*** **(%)**	359 (17%)	151 (10%)	208 (32%)
No	927 (43%)	565 (37%)	362 (56%)
Unknown	872 (40%)	792 (53%)	80 (12%)
**CHF,** ***n*** **(%)**	11 (0.5%)	1 (<0.1%)	10 (1.5%)
No	1273 (59%)	713 (47%)	560 (86%)
Unknown	874 (41%)	794 (53%)	80 (12%)
**Haemoglobinopathy,** ***n*** **(%)**	8 (0.4%)	3 (0.2%)	5 (0.8%)
No	1276 (59%)	711 (47%)	565 (87%)
Unknown	874 (41%)	794 (53%)	80 (12%)
**HIV,** ***n*** **(%)**	2 (<0.1%)	1 (<0.1%)	1 (0.2%)
No	1281 (59%)	713 (47%)	568 (87%)
Unknown	875 (41%)	794 (53%)	81 (12%)
**COPD/Asthma,** ***n*** **(%)**	219 (10%)	98 (6.5%)	121 (19%)
No	999 (46%)	576 (38%)	423 (65%)
Unknown	940 (44%)	834 (55%)	106 (16%)
**CKD,** ***n*** **(%)**	79 (3.7%)	32 (2.1%)	47 (7.2%)
No	1189 (55%)	674 (45%)	515 (79%)
Unknown	890 (41%)	802 (53%)	88 (14%)
**Liver Disease,** ***n*** **(%)**	94 (4.4%)	51 (3.4%)	43 (6.6%)
No	1190 (55%)	663 (44%)	527 (81%)
Unknown	874 (41%)	794 (53%)	80 (12%)
**b/ts DMARD Treatment**			
**TNF,** ***n*** **(%)**	1456 (67%)	1084 (72%)	372 (57%)
**IL-17,** ***n*** **(%)**	126 (5.8%)	97 (6.4%)	29 (4.5%)
**JAK,** ***n*** **(%)**	146 (6.8%)	95 (6.3%)	51 (7.8%)
**IL-6,** ***n*** **(%)**	81 (3.8%)	47 (3.1%)	34 (5.2%)
**IL-23,** ***n*** **(%)**	11 (0.5%)	7 (0.5%)	4 (0.6%)
**Other b/tsDMARD,** ***n*** **(%)**	57 (2.6%)	42 (2.8%)	15 (2.3%)
**Vaccination**			
**Influenza vaccination,** ***n*** **(%)**	625 (29%)	219 (15%)	406 (62%)
**Shingle vaccination,** ***n*** **(%)**	261 (12%)	64 (4.2%)	197 (30%)
**COVID-19 vaccination,** ***n*** **(%)**	1151 (53%)	524 (35%)	627 (96%)

Legend: RA: Rheumatoid arthritis; AS: Ankylosing spondylitis; PSA: psoriatic arthritis; AxSp: axial spondyloarthropathy; IA: inflammatory arthritis; IHD: ischemic heart disease; CHF: congestive heart failure; HIV: Human Immunodeficiency virus; COPD: chronic obstructive pulmonary disease; CKD: chronic kidney disease; b/ts: biologic or targeted synthetic; TNF: tumour necrosis factor; IL-17: interleukin-17; JAK: Janus kinase; IL-6: interleukin-6; IL-23: interleukin-23; DMARD: Disease-modifying anti-rheumatic drugs.

**Table 2:** Uptake of pneumococcal, influenza, and COVID vaccines.

	Pneumococcal Vaccine Uptake		
	Total (*n* = 2158)	Yes (*n* = 650)	No (*n* = 1508)
**Influenza vaccine in the last year,** ***n*** **(%)**			
Yes	625 (29.4%)	406 (62.5%)	219 (14.5%)
No	1533 (71.0%)	244 (37.5%)	1289 (85.5%)
**COVID-19 vaccine,** ***n*** **(%)**			
Yes	1151 (53.3%)	627 (96.5%)	524 (34.7%)
No	1007 (46.7%)	23 (3.5%)	984 (65.3%)

**Table 3:** Estimates from Poisson regression models assessing factors associated with pneumococcal vaccination.

Variable	Category	Risk Ratio (95% CI)	Adjusted Risk Ratio (95% CI)
**Age group**	<45(reference)	-	
	45–64	1.69 (1.36–2.11)	1.35 (1.08–1.69)
	≥65	2.66 (2.16–3.27)	1.92 (1.52–2.42)
**Gender**	Male	0.84 (0.73–0.98)	0.91 (0.79–1.05)
**Ethnicity**	White (reference)	-	
	Asian	0.91 (0.74–1.13)	0.96 (0.77–1.18)
	Black	0.87 (0.54–1.41)	0.97 (0.61–1.54)
	Mixed	0.40 (0.17–0.97)	0.49 (0.20–1.22)
	Other	0.83 (0.47–1.49)	0.85 (0.49–1.49)
**Any at-risk condition**	Yes	1.72 (1.46–2.02)	1.42 (1.20–1.69)
**b/tsDMARD exposure**	Yes	0.53 (0.47–0.61)	0.55 (0.48–0.63)
**Inflammatory arthritis subtype**	RA (reference)	-	
	AS	0.65 (0.52–0.82)	0.84 (0.67–1.05)
	PSA	0.85 (0.72–1.01)	1.00 (0.84–1.19)
	AxSp	0.57 (0.32–1.00)	0.78 (0.45–1.33)
	Undifferentiated IA	0.85 (0.55–1.32)	0.99 (0.65–1.50)

Legend RA: Rheumatoid arthritis; AS: Ankylosing spondylitis; PSA: psoriatic arthritis; AxSp: axial spondyloarthropathy; IA: inflammatory arthritis; b/tsDMARD: biologic or targeted synthetic disease modifying anti-rheumatic drugs.

## Data Availability

The data presented in the study are available on request from the corresponding authors in conjunction with persmission from University Hospitals Birmingham NHS Trust.

## Supplementary Materials

The following supporting information can be downloaded at: https://www.mdpi.com/article/10.3390/vaccines14050400/s1, Table S1: Baseline table in patient <65 years.

## References

[1] Mehta B., Pedro S., Ozen G., Kalil A., Wolfe F., Mikuls T., Michaud K. (2019). Serious infection risk in rheumatoid arthritis compared with non-inflammatory rheumatic and musculoskeletal diseases: A US national cohort study. RMD Open.

[2] Pollard A.J., Bijker E.M. (2021). A guide to vaccinology: From basic principles to new developments. Nat. Rev. Immunol..

[3] Bechman K., Song K., Abhishek A., Adas M., Ahmed A., Bray L., Davidson A., Deepak S., Dey M., De Vere H. (2025). The 2025 British Society for Rheumatology guideline for the prescription and monitoring of conventional synthetic disease-modifying anti-rheumatic drugs. Rheumatology.

[4] Furer V., Rondaan C., Heijstek M.W., Agmon-Levin N., van Assen S., Bijl M., Breedveld F.C., D’Amelio R., Dougados M., Kapetanovic M.C. (2020). 2019 update of EULAR recommendations for vaccination in adult patients with autoimmune inflammatory rheumatic diseases. Ann. Rheum. Dis..

[5] Bass A.R., Chakravarty E., Akl E.A., Bingham C.O., Calabrese L., Cappelli L.C., Johnson S.R., Imundo L.F., Winthrop K.L., Arasaratnam R.J. (2023). 2022 American College of Rheumatology Guideline for Vaccinations in Patients With Rheumatic and Musculoskeletal Diseases. Arthritis Care Res..

[6] Callaghan T., Washburn D., Goidel K., Nuzhath T., Spiegelman A., Scobee J., Moghtaderi A., Motta M. (2022). Imperfect messengers? An analysis of vaccine confidence among primary care physicians. Vaccine.

[7] Ricci L., Fery C., Tubach F., Agrinier N., Gagneux-Brunon A. (2025). Health care institutions and their physicians are the greatest promoters of COVID-19 vaccine acceptance among health care workers. Vaccine.

[8] Barry E.V.H., Suffel A.M., Walker J., Andrews N., Campbell C.N.J., Goudie R., de Lusignan S., Leston M., Langan S.M., Stowe J. (2026). Disparities in uptake of Shingrix(R) vaccine in immunosuppressed individuals in England: A population-based cohort study. Vaccine.

[9] Lal H., Cunningham A.L., Godeaux O., Chlibek R., Diez-Domingo J., Hwang S.J., Levin M.J., McElhaney J.E., Poder A., Puig-Barberà J. (2015). Efficacy of an adjuvanted herpes zoster subunit vaccine in older adults. N. Engl. J. Med..

[10] Pier M., Wolbink G., Boekel L. (2024). Time to talk to adults with rheumatic diseases about herpes zoster vaccination. Lancet Rheumatol..

[11] Mayor S. (2016). Shingles vaccine coverage for 70 year olds is falling. BMJ.

[12] Kim Y.H., Hong K.J., Kim H., Nam J.H. (2022). Influenza vaccines: Past, present, and future. Rev. Med. Virol..

[13] Pereira M., Williams S., Restrick L., Cullinan P., Hopkinson N.S., London Respiratory N. (2017). Healthcare worker influenza vaccination and sickness absence—An ecological study. Clin. Med..

[14] Kasstan B., Lazarus R., Ali I., Mounier-Jack S. (2024). Improving influenza vaccine uptake in clinical risk groups: Patient, provider and commissioner perspectives on the acceptability and feasibility of expanding delivery pathways in England. BMJ Public Health.

[15] Nagra D., Bechman K., Russell M.D., Yang Z., Adas M.A., Molabanti H.K., Molabanti H.K., Khan A., Wincup C., Alveyn E. (2025). Pneumococcal vaccine uptake in patients with inflammatory arthritis: A single centre cohort study. Rheumatology.

[16] Auroux M., Fabacher T., Sauleau E., Arnaud L., Coury F. (2025). Pneumococcal and influenza vaccination coverage and impact on COVID-19 infection severity in patients with inflammatory rheumatic diseases: A French National Healthcare Database analysis. Vaccine.

[17] Destordeur L.C., Lopez Delhoulle V., Papadopoulos I., Maes N., Fombellida K., El Moussaoui M., Darcis G. (2025). Factors Contributing to Pneumococcal, COVID-19, and Influenza Vaccine Uptake Among People Living With HIV in Belgium: A Retrospective Study. Open Forum Infect. Dis..

[18] Nakafero G., Grainge M.J., Card T., Mallen C.D., Nguyen Van-Tam J.S., Abhishek A. (2025). Uptake and safety of pneumococcal vaccination in adults with immune-mediated inflammatory diseases: A UK wide observational study. Rheumatology.

[19] Tan P.S., Patone M., Clift A.K., Dambha-Miller H., Saatci D., Ranger T.A., Garriga C., Zaccardi F., Shah B.R., Coupland C. (2023). Factors influencing influenza, pneumococcal and shingles vaccine uptake and refusal in older adults: A population-based cross-sectional study in England. BMJ Open.

[20] Liu B., Zhang X., Lai Y., Sun T., Wang C., Zhao T., Zhang S., Shi B., Li Y., Cui F. (2025). Global vaccine confidence trends among adults above and below age 65. npj Vaccines.

[21] Furer V., Weil C., Chodik G., Slav S.A., Blonder S.N., Fisher-Shoval Y., Barak M., Elkayam O. (2024). Real-World Coverage With Influenza, Pneumococcal, and Herpes Zoster Vaccines Among Patients With Rheumatic Diseases in a Nationwide Healthcare Plan. J. Rheumatol..

[22] Livieratos A., Gogos C., Akinosoglou K. (2024). Impact of Prior COVID-19 Immunization and/or Prior Infection on Immune Responses and Clinical Outcomes. Viruses.

[23] Radhakrishnan S.T., Perry R., Misra S., Ray S., Ruban A., Quayson B.I., Fofaria R., Hudovsky A., Williams H.R. (2024). Targeted education for clinicians and clinical coding staff improves the accuracy of clinical coding: A quality improvement project. Future Healthc. J..

[24] Craig L. (2018). Service improvement in health care: A literature review. Br. J. Nurs..

[25] Bosaeed M., Kumar D. (2018). Seasonal influenza vaccine in immunocompromised persons. Hum. Vaccines Immunother..

[26] Remane Y., Klaus V.C., Heinitz K., Ranft D., Kowald J., Herber A., Tautenhahn H.-M., Bertsche T., Ziganshyna S. (2025). Assessment of vaccination rates and motivation among transplant patients using vaccination cards and interviews. Sci. Rep..

[27] Lasagna A., Alessio N., Gambini G., Klersy C., Monaco T., Corallo S., Cicognini D., Pedrazzoli P. (2024). Vaccine hesitancy in patients with solid tumors: A cross-sectional single-center survey. BMC Public Health.

[28] Butow P., Shaw J., Bartley N., Milch V., Sathiaraj R., Turnbull S., Der Vartanian C. (2023). Vaccine hesitancy in cancer patients: A rapid review. Patient Educ. Couns..

[29] Felzer J.R., Finney Rutten L.J., Wi C.I., LeMahieu A.M., Beam E., Juhn Y.J., Jacobson R.M., Kennedy C.C. (2023). Disparities in vaccination rates in solid organ transplant patients. Transpl. Infect. Dis..

[30] Dendle C., Stuart R.L., Mulley W.R., Holdsworth S.R. (2018). Pneumococcal vaccination in adult solid organ transplant recipients: A review of current evidence. Vaccine.

[31] Holzer L., Hoffman T., Van Kessel D.A., Rijkers G.T. (2020). Pneumococcal vaccination in lung transplant patients. Expert Rev. Vaccines.

[32] Rodziewicz M., Dyball S., Bruce I., Parker B. (2021). Pausing drugs and spacing vaccines: An open question. Lancet Rheumatol..

[33] Kim Y., Taniguchi H., Okuyama K., Kawakami K. (2025). Coverage rates and reasons for pneumococcal vaccination among adults with chronic medical conditions and the elderly in Japan: A web-based, cross-sectional study. BMJ Open.

[34] Kafadar A.H., Sabatini S., Jones K.A., Dening T. (2024). Categorising interventions to enhance vaccine uptake or reduce vaccine hesitancy in the United Kingdom: A systematic review and meta-analysis. Vaccine.

[35] Liu S., Durantini M.R., Calabrese C., Sanchez F., Albarracin D. (2024). A systematic review and meta-analysis of strategies to promote vaccination uptake. Nat. Hum. Behav..

[36] Vojtek I., van Wouw M., Thomson A. (2024). Impact of COVID-19 on vaccine confidence and uptake: A systematic literature review. Hum. Vaccines Immunother..

[37] Dyer C. (2023). Patients launch legal action against AstraZeneca over its COVID-19 vaccine. BMJ.

[38] Sutton T. (2025). Annual flu vaccines are far from ideal—This is why. Nature.

[39] Lorenc T., Marshall D., Wright K., Sutcliffe K., Sowden A. (2017). Seasonal influenza vaccination of healthcare workers: Systematic review of qualitative evidence. BMC Health Serv. Res..

[40] Ferragut M.J., Barry D., Cummins M. (2020). Understanding why healthcare workers refuse the flu vaccine. J. Infect. Prev..

[41] Rimmer A. (2021). Do doctors have to have the covid-19 vaccine?. BMJ.

[42] Wojczewski S., Leitner K.M., Hoffmann K., Kutalek R., Jirovsky-Platter E. (2024). Vaccine hesitancy among physicians: A qualitative study with general practitioners and paediatricians in Austria and Germany. BMJ Open.

[43] Zastawna B., Zaluska R., Milewska A., Zdeba-Mozola A., Ogonowska A., Kozlowski R., Owczarek A., Marczak M. (2023). Protective Vaccination Used by Doctors for Prevention of Infections. Int. J. Environ. Res. Public Health.

[44] Martin Perez C., Ruiz-Rius S., Ramirez-Morros A., Vidal M., Opi D.H., Santamaria P., Blanco J., Vidal-Alaball J., Beeson J.G., Molinos-Albert L.M. (2025). Post-vaccination IgG4 and IgG2 class switch associates with increased risk of SARS-CoV-2 infections. J. Infect..

[45] Siebner A.S., Griesbaum J., Huus K.E., Flugge J., Hopfensperger K., Michel T., Schneiderhan-Marra N., Sauter D., Kremsner P.G., Ley R.E. (2025). Class switch toward IgG2 and IgG4 is more pronounced in BNT162b2 compared to mRNA-1273 COVID-19 vaccinees. Int. J. Infect. Dis..

[46] Aurelia L.C., Purcell R.A., Theisen R.M., Kelly A., Esterbauer R., Ramanathan P., Lee W.S., Wines B.D., Hogarth P.M., Juno J.A. (2025). Increased SARS-CoV-2 IgG4 has variable consequences dependent upon Fc function, Fc receptor polymorphism, and viral variant. Sci. Adv..

[47] McKee C., Bohannon K. (2016). Exploring the Reasons Behind Parental Refusal of Vaccines. J. Pediatr. Pharmacol. Ther..

[48] Ugrak U., Aksungur A., Akyuz S., Sen H., Seyhan F. (2025). Understanding the rise of vaccine refusal: Perceptions, fears, and influences. BMC Public Health.

[49] Welch V.L., Metcalf T., Macey R., Markus K., Sears A.J., Enstone A., Langer J., Srivastava A., Cane A., Wiemken T.L. (2023). Understanding the Barriers and Attitudes toward Influenza Vaccine Uptake in the Adult General Population: A Rapid Review. Vaccines.

[50] Esposito S., Bianchini S., Tagliabue C., Umbrello G., Madini B., Di Pietro G., Principi N. (2018). Impact of a website based educational program for increasing vaccination coverage among adolescents. Hum. Vaccines Immunother..

[51] Kaufman J., Overmars I., Fong J., Tudravu J., Devi R., Volavola L., Vodonaivalu L., Jenkins K., Leask J., Seale H. (2024). Training health workers and community influencers to be Vaccine Champions: A mixed-methods RE-AIM evaluation. BMJ Glob. Health.

[52] Pelullo C.P., Corea F., Della Polla G., Napolitano F., Di Giuseppe G. (2022). Schoolteachers and Vaccinations: A Cross-Sectional Study in the Campania Region. Vaccines.

[53] Kwella H., Schilbert J., Tessartz A., Scheersoi A. (2025). From classrooms to real-world contexts: Enhancing vaccine education through open schooling. Front. Public Health.

