# Supplementary material for: Uptake of Shingles, Influenza, COVID-19 and Pneumococcal Vaccination in Patients with Inflammatory Arthritis: A Three-Centre Study
Source: Vaccines (Basel). 2026 Apr 29;14(5):400. doi: 10.3390/vaccines14050400 (PMC13211440; doi:10.3390/vaccines14050400)
Supplement: Supplementary file 1 [file vaccines-14-00400-s001.zip › vaccines-4214667-supplementary.pdf]

Table S1. Baseline table in patient <65 years.

Characteristic	Overall N = 1,086	Pneumococcal vaccination No N = 730	Pneumococcal vaccination Yes N = 356
**Age**	**51 (40, 58)**	**48 (39, 57)**	**54 (44, 59)**
**Gender-Female, n (%)**	670 (62%)	438 (60%)	232 (65%)
**Ethnicity, n (%)**			
White	493 (45%)	303 (42%)	190 (53%)
Asian	144 (13%)	91 (12%)	53 (15%)
Black	24 (2.2%)	16 (2.2%)	8 (2.2%)
Mixed	16 (1.5%)	13 (1.8%)	3 (0.8%)
Other	20 (1.8%)	14 (1.9%)	6 (1.7%)
Unknown	389 (36%)	293 (40%)	96 (27%)
**Inflammatory arthritis, n (%)**			
RA	563 (52%)	364 (50%)	199 (56%)
AS	208 (19%)	160 (22%)	48 (13%)
PSA	247 (23%)	156 (21%)	91 (26%)
AxSp	34 (3.1%)	26 (3.6%)	8 (2.2%)
Undifferentiated IA	34 (3.1%)	24 (3.3%)	10 (2.8%)
**At-risk condition (any), n (%)**	364 (34%)	202 (28%)	162 (46%)
**Cancer, n (%)**	37 (3.4%)	26 (3.6%)	11 (3.1%)
No	820 (76%)	515 (71%)	305 (86%)
Unknown	229 (21%)	189 (26%)	40 (11%)
**Diabetes, n (%)**	95 (8.7%)	45 (6.2%)	50 (14%)
No	758 (70%)	492 (67%)	266 (75%)
Unknown	233 (21%)	193 (26%)	40 (11%)
**IHD, n (%)**	29 (2.7%)	16 (2.2%)	13 (3.7%)
No	829 (76%)	526 (72%)	303 (85%)
Unknown	228 (21%)	188 (26%)	40 (11%)
**Hypertension, n (%)**	164 (15%)	88 (12%)	76 (21%)
No	695 (64%)	455 (62%)	240 (67%)
Unknown	227 (21%)	187 (26%)	40 (11%)
**CHF, n (%)**	3 (0.3%)	1 (0.1%)	2 (0.6%)
No	856 (79%)	542 (74%)	314 (88%)
Unknown	227 (21%)	187 (26%)	40 (11%)
**Haemoglobinopathy, n (%)**	6 (0.6%)	3 (0.4%)	3 (0.8%)
No	853 (79%)	540 (74%)	313 (88%)
Unknown	227 (21%)	187 (26%)	40 (11%)
**HIV, n (%)**	1 (<0.1%)	1 (0.1%)	0 (0%)
No	857 (79%)	542 (74%)	315 (88%)
Unknown	228 (21%)	187 (26%)	41 (12%)
**COPD/Asthma, n (%)**	128 (12%)	70 (9.6%)	58 (16%)
No	676 (62%)	436 (60%)	240 (67%)
Unknown	282 (26%)	224 (31%)	58 (16%)
**CKD, n (%)**	28 (2.6%)	14 (1.9%)	14 (3.9%)
No	820 (76%)	523 (72%)	297 (83%)
Unknown	238 (22%)	193 (26%)	45 (13%)
**Liver Disease, n (%)**	60 (5.5%)	40 (5.5%)	20 (5.6%)
No	799 (74%)	503 (69%)	296 (83%)
Unknown	227 (21%)	187 (26%)	40 (11%)

Characteristic	Overall N = 1,086	Pneumococcal vaccination No N = 730	Pneumococcal vaccination Yes N = 356
**Vaccination**			
**Influenza vaccination, n (%)**	327 (30%)	136 (19%)	191 (54%)
**Shingle vaccination, n (%)**	90 (8.3%)	26 (3.6%)	64 (18%)
**COVID-19 vaccination, n (%)**	736 (68%)	398 (55%)	338 (95%)
**b/ts DMARD Treatment**			
**TNF, n (%)**	696 (64%)	498 (68%)	198 (56%)
**IL17, n (%)**	70 (6.4%)	45 (6.2%)	25 (7.0%)
**JAK, n (%)**	72 (6.6%)	41 (5.6%)	31 (8.7%)
**IL6, n (%)**	31 (2.9%)	24 (3.3%)	7 (2.0%)
**IL23, n (%)**	9 (0.8%)	5 (0.7%)	4 (1.1%)
**Other btsDMARD, n (%)**	16 (1.5%)	10 (1.4%)	6 (1.7%)

[1]n (%); Median (Q1, Q3)
